# Using standard and institutional mentorship models to implement SLMTA in Kenya

**DOI:** 10.4102/ajlm.v3i2.220

**Published:** 2014-11-03

**Authors:** Ernest P. Makokha, Samuel Mwalili, Frank L. Basiye, Clement Zeh, Wilfred I. Emonyi, Raphael Langat, Elizabeth T. Luman, Jane Mwangi

**Affiliations:** 1 Division of Global HIV/AIDS, US Centers for Disease Control and Prevention, Kenya; 2 Academic Model Providing Access to Healthcare (AMPATH), Moi University School of Medicine, Kenya; 3 Henry Jackson Foundation, Kenya; 4 International Laboratory Branch, Division of Global HIV/AIDS, US Centers for Disease Control and Prevention, United States

## Abstract

**Background:**

Kenya is home to several high-performing internationally-accredited research laboratories, whilst most public sector laboratories have historically lacked functioning quality management systems. In 2010, Kenya enrolled an initial eight regional and four national laboratories into the Strengthening Laboratory Management Toward Accreditation (SLMTA) programme. To address the challenge of a lack of mentors for the regional laboratories, three were paired, or ‘twinned’, with nearby accredited research laboratories to provide institutional mentorship, whilst the other five received standard mentorship.

**Objectives:**

This study examines results from the eight regional laboratories in the initial SLMTA group, with a focus on mentorship models.

**Methods:**

Three SLMTA workshops were interspersed with three-month periods of improvement project implementation and mentorship. Progress was evaluated at baseline, mid-term, and exit using the Stepwise Laboratory Quality Improvement Process Towards Accreditation (SLIPTA) audit checklist and scores were converted into a zero- to five-star scale.

**Results:**

At baseline, the mean score for the eight laboratories was 32%; all laboratories were below the one-star level. At mid-term, all laboratories had measured improvements. However, the three twinned laboratories had increased an average of 32 percentage points and reached one to three stars; whilst the five non-twinned laboratories increased an average of 10 percentage points and remained at zero stars. At exit, twinned laboratories had increased an average 12 additional percentage points (44 total), reaching two to four stars; non-twinned laboratories increased an average of 28 additional percentage points (38 total), reaching one to three stars.

**Conclusion:**

The partnership used by the twinning model holds promise for future collaborations between ministries of health and state-of-the-art research laboratories in their regions for laboratory quality improvement. Where they exist, such laboratories may be valuable resources to be used judiciously so as to accelerate sustainable quality improvement initiated through SLMTA.

## Introduction

Medical research laboratories are scattered globally in order to support drug testing and development, epidemiologic and clinical studies, and emergency needs. These laboratories are typically well-funded state-of-the-art facilities with expert staff and well-maintained equipment. In Kenya, there are five high-performing research laboratories accredited to international standards. Funded by various international organisations, these laboratories are located in different regions of the country where they conduct donor-driven research. By contrast, as of the end of 2012, none of Kenya’s 300 public sector clinical laboratories were accredited to international standards.^1^ Kenya’s picture is representative of most of the developing world where public sector laboratories have suffered many years of neglect and operate without functioning quality management systems.^2,3^ As a result, a majority of these laboratories are persistent underperformers with regard to disease surveillance and outbreak investigation.^4^ In some clinical settings, unreliable and inaccurate laboratory services continue to discourage healthcare providers from using laboratory testing to support disease diagnosis.^5^ This practice may delay the realisation of the United Nation’s Millennium Development Goals, as quality laboratory testing is critical for medical diagnostics, care and treatment of diseases.^6^

In the last several years, because of resources provided primarily by the US President’s Emergency Plan for AIDS Relief (PEPFAR) and its strategic partners, public sector laboratories in Kenya are on the mend. For instance, Kenya was amongst the first African countries to develop national laboratory policy guidelines and a five-year national strategic plan in an effort to improve the overall quality of laboratory services to meet the rising demand for HIV testing and treatment monitoring.^7^ Developed with support from PEPFAR, this strategic plan addressed a wide range of laboratory strengthening needs, including establishment of quality systems and local and international external quality assessment (EQA) programmes.^8^ These developments built the foundation for laboratory quality system improvements and enabled the country to roll out the Strengthening Laboratory Management Toward Accreditation (SLMTA) programme in April 2010, nine months after its launch in Kigali, Rwanda in 2009.^3^

Since the launch of SLMTA, observational studies have suggested that the mentorship component, especially when aligned to laboratory accreditation goals and overall plans of the Ministry of Health (MOH), provides substantial impact on laboratory quality improvement.^9^ SLMTA mentorship approaches have ranged from standard short-term visits^10,11^ to newer models of peer or embedded mentoring.^11,12^ In the standard short-term model, a mentor visits the mentee laboratory and stays in the laboratory for a short stint, often a week or less. During this period, the laboratory quality officer works under the guidance of the mentor to address laboratory quality gaps identified during prior assessment. This standard approach is used by most countries because it requires few staff and is thus low in cost, especially if in-country mentors are used. However, short visits may limit the amount of knowledge and skills transferred from the mentor to the mentee, as well as behavioural changes, both of which are critical requirements for successful transformation of quality systems practices in the laboratory. On the other hand, peer or embedded mentoring involves a mentor being in a laboratory for an extended duration, often weeks or months at a time. This enables the mentor to better understand the practices and personalities of the mentee laboratory and promotes positive changes in processes and behaviours.^9^ Both of these approaches are dependent on continuous availability of laboratory experts to serve as mentors. In much of sub-Saharan Africa, including Kenya, there is a generalised shortage of laboratory workforce;^13^ thus, maintaining a cohort of mentors to cover the ever-increasing number of laboratories in the region enrolling in SLMTA is a challenge.

This challenge was brought to the fore in Kenya where, through PEPFAR support, the first cohort of 12 high-volume laboratories – four national level and eight regional-level – began SLMTA implementation in 2010. With only six trained laboratory mentors available to cover the wide geographical spread of laboratories and to make repeated visits to the SLMTA laboratories, Kenya devised a multi-pronged mentorship approach for the 12 laboratories. The four national laboratories in Nairobi received comprehensive multi-partner technical support in an effort to achieve international standards of accreditation. For the eight regional laboratories, five were assisted by the six available mentors through standard short-term visitations as per the SLMTA guidelines. The remaining three regional laboratories received institutional mentorship (‘twinning’) through pairing with internationally-accredited high-performing research laboratories in their regions.

In this article, we focus on the eight regional laboratories where standard and institutional mentorship approaches were employed. We describe the institutional mentorship process and the improvements in laboratory quality systems in both twinned and non-twinned laboratories.

## Research methods and design

### SLMTA laboratories

**FIGURE 1:**
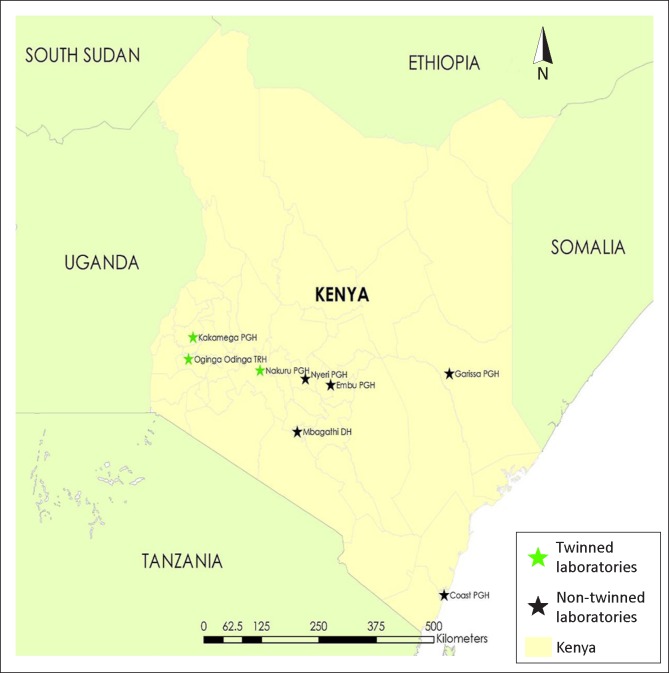
Map of Kenya showing the location of regional Strengthening Laboratory Management Toward Accreditation (SLMTA) cohort I laboratories.

The Kenya MOH purposively selected the eight regional laboratories to ensure geographical and regional balance (Figure 1). The selected laboratories are categorised as level V within Kenya’s national medical laboratory services policy,^7^ comprising seven provincial general hospital laboratories and one high-volume district hospital laboratory, all of which serve as regional hubs for EQA networks and back-up testing. The laboratories are functionally divided into seven testing departments: haematology, clinical chemistry, bacteriology/tuberculosis (TB) microscopy, blood transfusion, parasitology, histopathology/cytology and serology/virology/CD4. These departments are equipped with high-throughput equipment to support high-volume testing. Each testing bench has a technical head, typically a specialised laboratory technologist with a higher national diploma qualification.

All eight regional laboratories provide 24-hour service, seven days a week for specialised testing and other referral services to their regional catchment area, including CD4, clinical chemistries, HIV viral load and HIV early infant diagnosis. In addition, these laboratories serve as nodal points for split-sample quality assurance testing for district laboratories within their catchment region. All eight laboratories have infrastructure to allow for efficient workflow, such as running water and back-up power source.

At the onset of the SLMTA programme, all laboratories were participating in the following EQA schemes: the United Kingdom National EQA Scheme (NEQAS) for CD4 testing; the Human Quality Assessment Scheme (HuQAS) for clinical chemistry and haematology testing; national HIV Proficiency Testing for rapid HIV testing; malaria microscopy EQA provided by the East African Regional EQA Scheme (REQAS); and TB sputum microscopy provided by the Kenya National TB Laboratory. In addition, five of the laboratories were participating in the Quality Assessment and Standardization for Immunological Measures (QASI) CD4 testing scheme, with plans to cascade this service to the peripheral laboratories. All eight laboratories were using a paper-based laboratory information system at the beginning of the programme.

### SLMTA workshop series

In May 2010, the first four-day SLMTA workshop was conducted for representatives from each of the laboratories. After this workshop, the laboratories went through a three-month period of improvement project implementation and on-site mentorship. This was followed by a second SLMTA workshop in September 2010, followed by another round of improvement project implementation and on-site mentorship. The final SLMTA workshop was held in January 2011.

### Mentorship models

Two mentorship models were employed for the eight regional laboratories: the standard mentorship approach in five laboratories; and institutional twinning in three. The standard mentorship model is described in detail elsewhere^14^ and is based on mentors spending periods of time in laboratories to guide and oversee the quality systems improvement process. Six in-country, practising laboratory professionals were trained as mentors and completed the SLMTA Training-of-Trainers curriculum. Five were assigned to one laboratory each, whilst the sixth was tasked with overall coordination of mentorship activities. As an initial assignment, each mentor assisted their target laboratory to develop a three-month work plan, outlining SLMTA workshop topics, potential improvement projects and a plan for documentation of laboratory performance. Following each workshop, mentors visited their assigned laboratory once a month, with each visit lasting five days. At each visit, the mentor used the World Health Organization Regional Office for Africa (WHO AFRO) Stepwise Laboratory Quality Improvement Process Towards Accreditation (SLIPTA) checklist in order to measure and review improvements. In addition, the mentor worked with laboratory staff to accomplish a set of improvement projects identified during the SLMTA workshops.

For the institutional mentorship approach, the laboratory branch of the US Centers for Disease Control and Prevention (CDC), Kenya Office, contacted research laboratories to solicit their support for facility-based stepwise improvements of quality systems in targeted MOH laboratories. These laboratories are accredited to either International Organization for Standardization (ISO) or the College of American Pathologists (CAP) standards. Based on this agreement, each of the three regional MOH laboratories in western Kenya was twinned after the first SLMTA workshop with an accredited medical research laboratory operating in the same region (Table 1). This twinning relationship lasted for the duration of SLMTA programme. Through this partnership and under the guidance of the SLMTA coordinating mentor, research laboratories worked with the SLMTA laboratories to support the implementation of quality improvements through initial engagement and development of an action plan, documentation review and equipment assessment, training and on-site mentorship, exchange visits and performance review (Table 2).

**TABLE 1:** Accredited research laboratories and the public laboratories twinned with them through the Strengthening Laboratory Management Toward Accreditation (SLMTA) programme in western Kenya.

Research Laboratory	Main Tests	Accreditation (year)	Twin Laboratory
Kenya Medical Research Institute, HIV Research Laboratory Kisumu	Chemistries Hematology CD4 HIV Viral Load HIV DR TB microscopy and DST	ISO 15189 (2007)	Jaramogi Oginga Odinga Teaching and Referral Hospital (Formerly New Nyanza Provincial Hospital) Nyanza Province
Walter Reed Program, Research Laboratory Kericho	Chemistries Hematology CD4 HIV Viral Load HIV DR TB microscopy and DST	CAP (2008)	Nakuru Provincial Hospital Rift Valley Province
Moi University, School of Medicine, AMPATH Laboratory Eldoret	Chemistries Hematology CD4 HIV Viral Load HIV DR TB microscopy and DST	ISO 15189 (2010)	Kakamega Provincial Hospital Western Province

DR, drug resistance; TB, tuberculosis; DST, drug sensitivity testing; AMPATH, Academic Model Providing Access to Healthcare; ISO, International Organization for Standardization; CAP, College of American Pathologists.

**TABLE 2:** Institutional mentorship process.

Area of support	Description of activity
Initial engagement and action plan	Top-level managers of twinned laboratories met, agreed on the laboratory twinning concept, and identified individuals to serve as focal points. During this initial meeting, project timelines were established and a list of deliverables was developed.
Document review and equipment assessment	Research laboratory staff worked with SLMTA laboratory staff to review existing management, policy and standard operating procedure documents and developed new documents as needed. An equipment master list was prepared for each site.
Training and on-site mentorship	Facility-based trainings on good clinical laboratory practices, method validation, and internal audit were conducted by the research laboratory staff. On-site targeted mentorship was provided to all SLMTA laboratory staff, based on non-conformance findings from baseline audit. Specific mentorship subjects included document and records management, equipment management, and information management. On-site mentorship also included hands-on training in technical processes, biosafety, fire safety, and laboratory audits.
Exchange visits	Exchange visits were conducted in which SLMTA laboratory personnel visited the research laboratories for periods averaging five days to address needs identified in the baseline audits. All the mentee laboratory staff took turns visiting the research laboratory so as not to interrupt testing services at the laboratory. During these visits, the visiting staff worked at the accredited laboratory under the supervision of the quality assurance staff to gain hands-on experience in the following activities: organizing laboratory facilities, designing personnel files, developing competency assessment tools, performing internal quality control and monitoring Levey-Jennings charts, running and troubleshooting external quality assessments, operating the electronic laboratory information system, using safety signage, and managing laboratory waste. On reciprocal visits, the research laboratory quality assurance staff assisted in implementation of best practices at the SLMTA laboratory.
Performance review	Each pair of twinned laboratories held weekly meetings to review progress on the various laboratory improvement projects and to facilitate communication with hospital upper management. These meetings were co-chaired by the mentor and SLMTA laboratory manager and attended by all laboratory staff from the mentee laboratory.

SLMTA, Strengthening Laboratory Management Toward Accreditation.

### Ethical considerations

The multi-country SLMTA programme was approved by the CDC as non-human research. Under this approval, CDC staff provided technical support for work that did not involve possession or analysis of identifiable data or interaction with participants’ data.

### Baseline, mid-term and exit audits

SLMTA progress is evaluated using the SLIPTA checklist.^15^ The SLIPTA framework provides stepwise recognition toward fulfilment of the ISO 15189/17025 standard.^3^ As per SLIPTA guidelines, scores for every laboratory are converted into a zero- to five-star scale, with minimums set at 55% for one star, 65% for two stars, 75% for three stars, 85% for four stars and 95% for five stars.

The SLIPTA checklist covers the 12 Quality System Essentials (QSEs) as defined by the Clinical and Laboratory Standards Institute,^16^ which can be grouped into three stages of the quality cycle.^17^ Resource management (pre-examination) tasks include organisation and personnel, equipment management, purchasing and inventory, and facilities and safety. Process management (examination) tasks include documents and records, client management, information management and process control. Improvement management (post-examination) tasks include management reviews, internal audit, corrective action and occurrence/incidence management.

In March to April 2010, three weeks after enrolment and before the first SLMTA workshop, baseline audits were conducted in all eight laboratories, using the SLIPTA checklist. The audits were conducted by a team of in-country auditors led by a visiting auditor from the American Society for Clinical Pathology (ASCP). In addition to the baseline audits, a rapid assessment of the services provided by these laboratories was conducted. Results from the baseline audit and rapid assessment provided a basis for developing facility-specific work plans. Mid-term audits were conducted six months after enrolment, after the second SLMTA workshop and corresponding improvement projects, also using the SLIPTA checklist; these audits were conducted by an independent institution, the Kenya Accreditation Service (KENAS). Following the final SLMTA workshop and improvement project period, about 13 months after the baseline audit, exit audits were conducted by KENAS auditors.

### Data analysis

Data from the baseline, mid-term and exit audits were collected and analysed using SAS software version 9.3 (SAS Institute Inc., Cary, NC 2012). Analysis focused on describing the results of the two mentorship approaches. SLIPTA scores and star ratings were calculated by summing all points across the 12 QSEs. In addition, we examined results for each QSE separately and computed an average score by quality cycle stage, weighing all QSEs in the cycle equally. Exploratory data analysis was conducted by computing descriptive statistics (i.e., percentages, means and medians); graphical representation of the QSE scores was created for the twinned and non-twinned laboratories.

## Results

At baseline, the eight laboratories had a mean score of 32% on the SLIPTA checklist.^15^ Scores ranged from 16% to 44%, with all laboratories below the one-star level (Table 3). The mean baseline score for twinned laboratories was 36% and for non-twinned laboratories, 30%.

**TABLE 3:** Summary of audit results at baseline, mid-term, and exit of the Strengthening Laboratory Management Toward Accreditation (SLMTA) programme, Kenya 2010.

Laboratory	Audit Scores^†^					
	Baseline audit		Mid-term audit		Exit audit	
	Score (%)	Star rating	Score (%)	Star rating	Score (%)	Star rating
**Twinned**						
Nyanza	44	0	85	3	90	4
Nakuru	35	0	60	1	73	2
Kakamega	29	0	67	2	78	3

Mean	36	-	68	-	80	-
Median	35	-	67	-	78	-

**Non-twinned**						
Embu	31	0	54	0	64	1
Coast	38	0	33	0	59	1
Mbagathi	32	0	47	0	74	2
Garissa	16	0	31	0	65	1
Nyeri	33	0	38	0	79	3

Mean	30	-	40	-	68	-
Median	32	-	38	-	65	-

† Based on the Stepwise Laboratory Quality Improvement Towards Accreditation (SLIPTA) checklist.

There were considerable improvements in all but one laboratory at mid-term audit, with twinned laboratories improving substantially more than non-twinned laboratories (mean 32 percentage points vs. 10 percentage points). All three twinned laboratories had reached at least one star whilst all non-twinned laboratories were still at zero stars. At exit audit, twinned laboratories improved an additional average 12 percentage points to 80%, with one laboratory each at 2, 3 and 4 stars. Non-twinned laboratories improved an additional average 28 percentage points to 68%, with three laboratories at one star, one at two stars and one at three stars.

**FIGURE 2:**
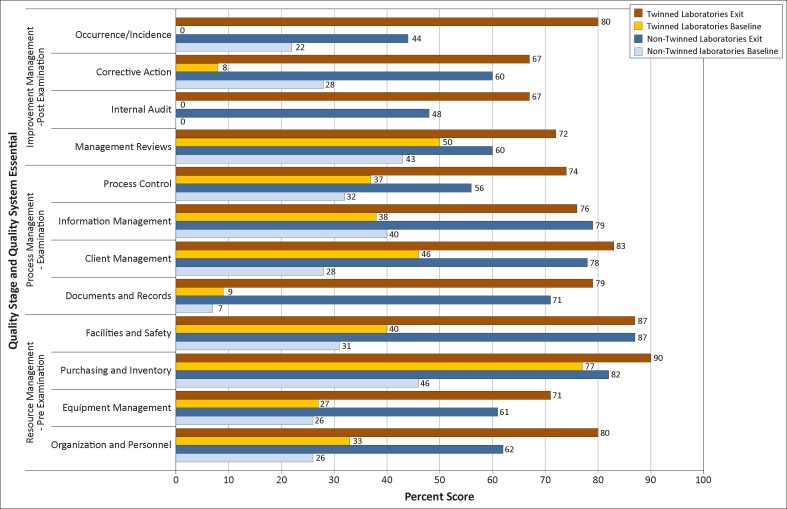
Performance on 12 Quality System Essentials for twinned and non-twinned laboratories at baseline and exit of the Strengthening Laboratory Management Toward Accreditation (SLMTA) programme.

**FIGURE 3:**
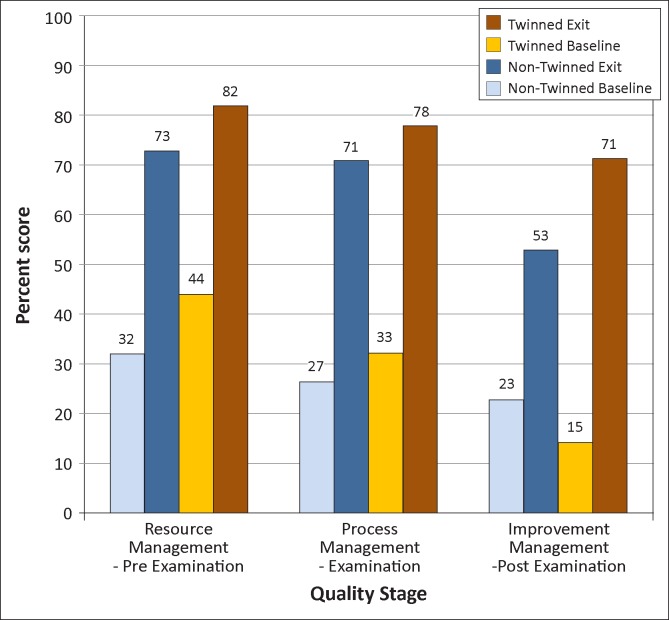
Average performance of twinned and non-twinned laboratories by quality cycle stages at baseline and exit of the Strengthening Laboratory Management Toward Accreditation (SLMTA) programme.

Figure 2 shows QSE results for the two sets of laboratories over the project period. For eight of the 12 QSEs, mean scores increased more amongst the twinned than non-twinned laboratories. The greatest improvements amongst the twinned laboratories were in the areas of occurrence/incidence management (from 0% to 80%), documents and records (from 9% to 79%) and internal audit (from 0% to 67%). Greatest improvements for non-twinned laboratories were in the areas of documents and records (from 7% to 71%), facilities and safety (from 31% to 87%) and client management (from 28% to 78%). Overall, laboratories scored highest in the resource management category of QSEs (73% for non-twinned and 82% for twinned laboratories at exit) and lowest in improvement management (53% and 71%, respectively) (Figure 3). Non-twinned laboratories improved slightly more than twinned laboratories in the resource management stage (41 percentage points vs. 38 percentage points), whereas twinned laboratories improved slightly more in the process management stage (44 percentage points vs. 45 percentage points) and substantially more in the improvement management stage (30 percentage points vs. 56 percentage points).

## Discussion

The eight regional-level laboratories in Kenya’s first SLMTA cohort all made substantial quality improvements, moving from zero SLIPTA stars to anywhere from one to four stars in a period of one year. Laboratories twinned with research institutions started slightly higher and improved nearly twice as much as non-twinned laboratories during the first half of the programme. However, after mid-term, non-twinned laboratories increased their efforts and nearly caught up with their twinned counterparts. By the exit audit, twinned laboratories had improved by 44 percentage points, whilst non-twinned laboratories had improved by 38 percentage points.

Twinning SLMTA laboratories with research institutions addressed several observed needs. Previous site assessment reports had documented a large disparity between government-funded and research-sponsored laboratories with regard to available equipment, supplies, quality assurance, safety practices and overall management. A consultancy team from the Association of Public Health Laboratories observed that there had previously been little to no sharing of resources and knowledge between the two groups, even where they were physically close in location;^18^ such was the case with the Kenya Medical Research Institute (KEMRI) HIV Research Laboratory, with state-of-the-art facilities and ISO accreditation since 2007, and the Nyanza Provincial General Hospital, which are located on the same campus. The twinning approach helped to bring the laboratories together to bridge the quality differential gap between the research laboratories and public laboratories that serve the majority of the population.

Twinning MOH laboratories with high-performing laboratories also helped to alleviate the shortage of SLMTA mentors. The approach gave the government-sponsored laboratories access to highly-skilled personnel with technical expertise from the research laboratories, who were able to both mentor and monitor their quality performance. In addition, institutional mentorship provided extended contact time and engaged the entire laboratory workforce in the twinned MOH laboratory, facilitating measurable QSE improvements across the laboratory testing system.

In the context of ongoing laboratory strengthening efforts^19^ and PEPFAR’s recent approaches to promoting public–private partnerships to enhance African countries’ laboratory systems and service delivery,^20^ Kenya’s twinning model is the first of its kind to create in-country laboratory partnerships in order to address not only quality systems, but also sustainable strategies for capacity building. Review of previous laboratory partnerships reveals two scenarios: the majority have been international partnerships focusing largely on vertical disease programmes and emerging global disease surveillance programmes,^21^ whilst the remainder focused on transfer of technologies in health and support of national scientists to apply for grants from local and international funding agencies.^22^ The results presented here provide evidence that institutional twinning of in-country high-performing laboratories with MOH counterparts through the SLMTA programme is a practical means of achieving quality improvements for public diagnostic laboratories in low-resource settings.

The exchange visits between the twinned laboratories provided the mentee laboratory staff additional motivation to adopt and implement quality systems in their laboratories. Through exchange visits, the mentee laboratories had opportunities to experience first-hand good laboratory practices and thereafter found them easier to implement at their own facilities. In addition to the rapid improvements achieved through twinning, periodic visits to the research laboratories by the mentee laboratory staff promoted an integrated understanding of laboratory quality systems and further enhanced quality services provided by the mentee laboratories.

In our study, twinned laboratories had the most advances in the improvement management category, which focuses mainly on post-examination tasks. These twinned laboratories did particularly well in occurrence and/or incidence management, internal audit and corrective action, all of which are critical for reducing preventable laboratory errors and hence ensuring that laboratories produce accurate results for quality patient care. In contrast, non-twinned laboratories improved more in the resource management and process management areas, which focus on pre-examination and examination tasks. These tasks require hospital management decisions and activities that not only support, but also enhance technical work performed by the laboratory. Within one year of mentorship, several improvements were recorded in the non-twinned laboratories, including the establishment of laboratory personnel files with job descriptions, an accessible system for laboratory records and documents, management review calendars and a clinician handbook. The two sets of laboratories recorded comparable improvements in the equipment management and management review QSEs. However, twinning was associated with better outcomes in the QSEs that typically require close communication between the laboratory and upper management for approval of decisions and budgetary needs.

Several factors may limit the generalisation of our observations. Firstly, the number of laboratories was very small, prohibiting statistical assessment of the difference in results between the twinned and non-twinned laboratories. Secondly, laboratories were not assigned randomly to their respective mentorship model, so any effects could have resulted from factors that also influenced assignment; however, the two sets of laboratories had identical SLMTA training and comparable mean baseline scores, both overall and in most categories. Thirdly, mentorship works through a positive rapport established between the mentor and mentee; despite use of a standardised checklist, personality differences amongst mentors and SLMTA laboratory staff members, as well as the greater contact time and frequent benchmarking visits for the twinned laboratories, may have affected the results. Finally, one of the MOH laboratories is located on the same campus as its twinned research laboratory; whilst this proximity has the advantage of very close monitoring and review of quality improvements, results may not be typical of what would be expected with more distant twinned laboratories.

Kenya’s institutional mentorship approach went beyond standard SLMTA training to include on-site provision of Good Clinical Laboratory Practice training. Through benchmarking and exchange visits, the MOH laboratories were able to observe, practise and develop personnel files, competency assessment tools, Levey-Jennings charts, quality control runs and EQA data tools. This institutional mentorship approach appears to have contributed to the rapid improvement in the twinned laboratories during the first half of the programme, as the MOH laboratory staff quickly put into practice what they had observed. The limited progress made during the second half of the programme amongst twinned laboratories suggests that further improvements may rely on external factors, such as additional funding and staff; and that institutional mentorship may be most efficient when used for short durations.

Public–private partnership models have been gaining popularity in recent years as a potential source of funding and technical support for improvement in public sector activities. The World Bank suggests that these partnerships may be a good way to build skills in the public sector.^23^ Balancing the need for support with the host government’s responsibility for providing services to the public is critical. Whilst the potential benefits are encouraging, it must be kept in mind that supporting public health laboratories is not the long-term responsibility of these research facilities; thus, care should be taken to identify ways to streamline the mentoring process and to use the programme as a catalyst for sustainable development of a permanent cadre of well-trained leaders within the public sector in order to ensure that the generosity of these research laboratories is maximised. Judicious use of this valuable resource and appropriate acknowledgement will help to ensure a mutually beneficial outcome, encouraging private laboratories to include partnership in their mandate as a means of fulfilling corporate social responsibility.

### Conclusion

The institutional twinning model holds promise for future collaborations between ministries of health and state-of-the-art local laboratories accredited to international standards. This model may be used to foster long-standing and sustainable partnerships between public health and research laboratories.

## References

[1] Schroeder LF, Amukele T Medical laboratories in sub-Saharan Africa that meet international quality standards. Am J Clin Pathol. 2014;141(6):791–795. http://dx.doi.org/10.1309/AJCPQ5KTKAGSSCFN 24838322 10.1309/AJCPQ5KTKAGSSCFN

[2] World Health Organization Joint WHO–CDC conference on health laboratory quality systems. WHO/HSE/IHR/LYO/2008.3 [document on the Internet]. c2008 [cited 2014 Mar 15]. Available from: http://www.who.int.ihr/lyon/report20080409.pdf

[3] Gershy-Damet GM, Rotz P, Cross D, et al The World Health Organization African region laboratory accreditation process: Improving the quality of laboratory systems in the African region. Am J Clin Pathol. 2010;134(3):393–400. http://dx.doi.org/10.1309/AJCPTUUC2V1WJQBM 20716795 10.1309/AJCPTUUC2V1WJQBM

[4] Nkengasong J, Nsubuga P, Nwanyanwu O, et al Laboratory systems and services are critical in global health: Time to end the neglect? Am J Clin Pathol. 2010;134(3):368–373. http://dx.doi.org/10.1309/AJCPMPSINQ9BRMU6 20716791 10.1309/AJCPMPSINQ9BRMU6 PMC7109802

[5] Petti CA, Polage CR, Quinn TC, et al Laboratory medicine in Africa: Barrier to effective health care. Clin Infect Dis. 2006;42(3):377–382. http://dx.doi.org/10.1086/499363 16392084 10.1086/499363

[6] Anyangwe SCE, Mtonga C, Chirwa B Health inequities, environmental insecurity and the attainment of the Millennium Development Goals in sub-Saharan Africa: The case study of Zambia. Int J Environ Res Public Health. 2006;3(3):217–227. http://dx.doi.org/10.3390/ijerph2006030026 16968967 10.3390/ijerph2006030026 PMC3807514

[7] Kenya Ministry of Health National policy guidelines for medical laboratory services. Nairobi; 2006 (Unpublished).

[8] Kenya Ministry of Health National Laboratory Services of Kenya. National Strategic Plan 2005–2010. Nairobi; 2005 (Unpublished).

[9] Maruta T, Motebang D, Wanyoike J, et al Impact of mentorship on WHO-AFRO Strengthening Laboratory Quality Improvement Process Towards Accreditation (SLIPTA). Afr J Lab Med. 2012;1(1), Art. #6, 8 pages. http://dx.doi.org/10.4102/ajlm.v1i1.6 10.4102/ajlm.v1i1.6 PMC5644515 29062726

[10] Nzabahimana I, Sebasirimu S, Gatabazi JB, et al Innovative strategies for a successful SLMTA country programme: The Rwanda story. Afr J Lab Med. 2014;3(2), Art. #217, 6 pages. http://dx.doi.org/10.4102/ajlm.v3i2.217 10.4102/ajlm.v3i2.217 PMC5637798 29043189

[11] Nzombe P, Luman ET, Shumba E, et al Maximising mentorship: Variations in laboratory mentorship models implemented in Zimbabwe. Afr J Lab Med. 2014;3(2), Art. #241, 8 pages. http://dx.doi.org/10.4102/ajlm.v3i2.241 10.4102/ajlm.v3i2.241 PMC5637805 29043196

[12] Audu RA, Onubogu CC, Nwokoye NN, et al Improving quality in national reference laboratories: The role of SLMTA and mentorship. Afr J Lab Med. 2014;3(2), Art. #200, 7 pages. http://dx.doi.org/10.4102/ajlm.v3i2.200 10.4102/ajlm.v3i2.200 PMC5637787 29043183

[13] Chankova S, Muchiri S, Kombe G Health workforce attrition in public sector in Kenya: A look at the reasons. Hum Resour Health. 2009;7:58 http://dx.doi.org/10.1186/1478-4491-7-58 19622140 10.1186/1478-4491-7-58 PMC2720908

[14] Maruta T, Rotz P, Trevor P Setting up a structured laboratory mentoring programme. Afr J Lab Med. 2013;2(1), Art. #77, 7 pages. 10.4102/ajlm.v2i1.77 PMC5637775 29043168

[15] World Health Organization Regional Office for Africa WHO guide for the stepwise laboratory improvement process towards accreditation in the African Region (with checklist) [document on the Internet]. c2011 [cited 2014 Mar 15]. Available from: http://www.afro.who.int/index.php?option=com_docman&task=doc_download&gid=8642&Itemid=2593

[16] CLSI Quality management system: A model for laboratory services; approved guideline – Fourth Edition. CLSI document GP26-A4 Wayne, PA: Clinical and Laboratory Standards Institute; 2011.

[17] CLSI Quality management system: Continual improvement; approved guideline – Third Edition. Wayne, PA: Clinical and Laboratory Standards Institute; 2012.

[18] Kenya GAP/PEPFAR laboratory visit. Full Report, Nairobi Kenya January 30 to February 11, 2005; 2005 (Unpublished).

[19] Olmsted SS, Moore M, Meili RC, et al Strengthening laboratory systems in resource-limited settings. Am J Clin Pathol. 2010;134(3):374–380. http://dx.doi.org/10.1309/AJCPDQOSB7QR5GLR 20716792 10.1309/AJCPDQOSB7QR5GLR

[20] PEPFAR New public–private partnership to strengthen laboratory systems [page on the Internet]. c2007 [cited 2014 Aug 08]. Available from: http://www.pepfar.gov/documents/organization/94561.pdf

[21] Gambel JM, Hibbs RG Jr U.S. military overseas medical research laboratories. Mil Med. 1996;161(11):638–645. 8961715

[22] Chandiwana S, Ornbjerg N Review of North-South and South-South cooperation and conditions necessary to sustain research capability in developing countries. J Health Popul Nutr. 2003;21(3):288–297. 14717574

[23] Yu D, Souteyrand Y, Banda MA, et al Investment in HIV/AIDS programs: Does it help strengthen health systems in developing countries? Global Health. 2008;4:8 http://dx.doi.org/10.1186/1744-8603-4-8 18796148 10.1186/1744-8603-4-8 PMC2556650

